# Reverse Vaccinology Integrated with Biophysics Techniques for Designing a Peptide-Based Subunit Vaccine for Bourbon Virus

**DOI:** 10.3390/bioengineering11111056

**Published:** 2024-10-23

**Authors:** Taghreed N. Almanaa

**Affiliations:** Department of Botany and Microbiology, College of Science, King Saud University, Riyadh 11451, Saudi Arabia; talmanaa@ksu.edu.sa

**Keywords:** reverse vaccinology, linkers, immune receptors, binding energies, molecular dynamics simulations

## Abstract

Despite the seriousness of the disease carried by ticks, little is known about the Bourbon virus. Only three US states have recorded human cases of Bourbon virus (BRBV) infection; in all cases, a tick bite was connected with the onset of the illness. The Bourbon virus (BRBV) belongs to the Orthomyxoviridae family and Thogotovirus genus, originating in the states of the US, i.e., Kansas, Oklahoma and Missouri. The growing rates of BRBV infections in various parts of the US highlight the necessity for a thorough analysis of the virus’s transmission mechanisms, vector types and reservoir hosts. Currently, there are no vaccines or efficient antiviral therapies to stop these infections. It is imperative to produce a vaccination that is both affordable and thermodynamically stable to reduce the likelihood of future pandemics. Various computational techniques and reverse vaccinology methodologies were employed to identify specific B- and T-cell epitopes. After thorough examination, the linker proteins connected the B- and T-cell epitopes, resulting in this painstakingly constructed vaccine candidate. Furthermore, 3D modeling directed the vaccine construct toward molecular docking to determine its binding affinity and interaction with TLR-4. Human beta-defensin was used as an adjuvant and linked to the N-terminus to boost immunogenicity. Furthermore, the C-IMMSIM simulation resulted in high immunogenic activities, with activation of high interferon, interleukins and immunoglobulin. The results of the in silico cloning process for *E. coli* indicated that the vaccine construct will try its utmost to express itself in the host, with a codon adaptation CAI value of 0.94. A net binding free energy of −677.7 kcal/mol obtained during docking showed that the vaccine has a high binding affinity for immunological receptors. Further validation was achieved via molecular dynamic simulations, inferring the confirmational changes during certain time intervals, but the vaccine remained intact to the binding site for a 100 ns interval. The thermostability determined using an RMSF score predicted certain changes in the mechanistic insights of the loop region with carbon alpha deviations, but no major changes were observed during the simulations. Thus, the results obtained highlight a major concern for researchers to further validate the vaccine’s efficacy using in vitro and in vivo approaches.

## 1. Introduction

Global public health is faced with significant problems from the rise and resurgence of infectious illnesses, which are exacerbated by urbanization, climate change and globalization [1]. Tick-borne diseases act as one of the ongoing public health issues, accountable for over 95% of vector-borne diseases in the US and imposing substantial economic burdens worldwide [2]. Despite their high fatality rates and severe prognosis, tick-borne infections pose challenges in detection and diagnosis because of their wide clinical presentation and the lack of accurate diagnostic tests [3]. Moreover, the increasing tick populations are leading to a rise in the occurrence of these diseases, demanding immediate attention from scholars, researchers, scientists and public health professionals. Efficient tick control approaches and improved medical treatments are essential to alleviate the influence of tick-borne diseases and protect human and animal health in affected regions worldwide [4]. The human appearance of new tick-borne viruses in areas with high vector populations and frequent human contact is a significant public health concern. One such virus, the Bourbon virus (BRBV), belonging to the *Orthomyxoviridae* family and *Thogotovirus* genus [5], was originally discovered in blood samples of a hospitalized male (50 years old) in 2014, following the tragic death of a citizen of Bourbon County, Kansas, US [6,7]. The Bourbon virus (BRBV) is defined by a variety of debilitating symptoms, such as nausea, fever, rash, low platelet count, low white blood cell count, low lymphocyte count and increased levels of aspartate transferase and alanine transferase. In its late stages, the BRBV disease is characterized by shock, pleural effusions and cardiac dysregulation. In fatal cases, the period between the onset of symptoms and death has been found to be between 11 and 24 days. The examination after the death of the index case revealed a decrease in the activity of acute bone marrow [8]. BRBV swiftly attracted attention because of its link to tick bites and its ability to potentially spread to humans. The symptoms of BRBV in non-human vertebrates are still unidentified [7,9]. Viruses belonging to this family possess filamentous or rounded (≈100–130 nm), enveloped, segmented negative-sense single-stranded RNA genomes (≈10–11 kb) comprising six genes. These genes encode viral polymerase basic protein 1 (PB-1), polymerase basic protein 2 (PB-2), a matrix protein, polymerase acidic protein (PA), a nucleoprotein and a surface glycoprotein [6,10]. 

All documented human cases of Bourbon virus (BRBV) infection have only been reported in three states of the US: Kansas, Oklahoma and Missouri. Each incidence involved a tick bite that was linked to the beginning of the disease [8]. The increased prevalence of BRBV infections in various regions of the US underscored the need for a comprehensive investigation into its transmission dynamics, vector species and potential reservoir hosts [10,11]. The virus has been observed in many regions of Africa, the Middle East and Europe, where it has spread to humans and animals via tick bites [12].

BRBV is likely spread to humans and other vertebrates by lone star ticks (*Amblyomma americanum*), which are prevalent in southcentral US [13]. Additionally, the Bourbon virus has been found in Asian long-horned ticks (*Haemaphysalis longicornis*) as of the year 2022 as a result of surveillance research that was carried out in Virginia, US [8,14]. White-tailed deer and raccoons are commonly exposed to BRBV, although birds are not involved [5]. Dogs, horses, deer and raccoons could be used as possible sentinels to monitor the distribution of BRBV and reduce human risk. These findings highlight the necessity for more research to establish the competency of mammalian species as hosts that amplify BRBV [5]. 

Developing vaccines in laboratories can be time-consuming, labor-intensive and costly, which can endanger patients. Conventional vaccine creation using weakened micro-organisms poses a danger of toxicity [15]. Utilizing T- and B-cell peptides to induce immunological responses is a potential new strategy. Subunit vaccination depends on T-cell identification of pathogens and subsequent immune reactions. B-cell epitopes, essential for the adaptive immune system, interact with antibodies [15,16]. Reverse vaccinology is one of the immunoinformatic strategies that predict future vaccine candidates, including those that come from different gene lineages. Certain proteins have been found to have high antigenicity through research, suggesting that they could be good candidates for vaccines. The assessment of potential biomarker candidates prior to costly preclinical or clinical research is made possible by immunoinformatic technologies. By employing immunoinformatics to investigate these proteins as potential vaccine candidates, more in vitro and in vivo studies may be possible, which will open up new avenues for potential preventive measures [17]. As the scope of the paper is computational, the main objective of the work is to provide a theoretical vaccine model for researchers to check the designed vaccine’s immune protective efficacy against the said pathogen in vivo. Reverse vaccinology (RV) has received more attention in recent years and has been used to identify vaccine proteins against different pathogens [18]. The RV approach was first applied to the bacterial pathogen Meningococcus B (MenB) and led to the licensed Bexsero vaccine [19], where RV played a significant role in screening for an antigen with the broadest bactericidal activity and ultimately resolved the long journey of MenB vaccine development. RV has also been applied to many other bacterial pathogens, including group A Streptococcus, antibiotic-resistant Staphylococcus aureus, Streptococcus pneumonia and Chlamydia. The efficacy of peptide- or subunit-based vaccines initially identified through a RV protocol has also been proven experimentally [20,21]. In this study, a RV approach was used to screen possible vaccine proteins against the Bourbon virus, identifying two proteins—the nucleoprotein and the polymerase subunit PA protein—as strong candidates for vaccine development. Experimental follow-up via testing of the immune protection efficacy of the screened epitopes in animal models will be open for researchers, and this study will speed up the vaccine development process against this pathogen. 

## 2. Materials and Methods

**Figure 1:**
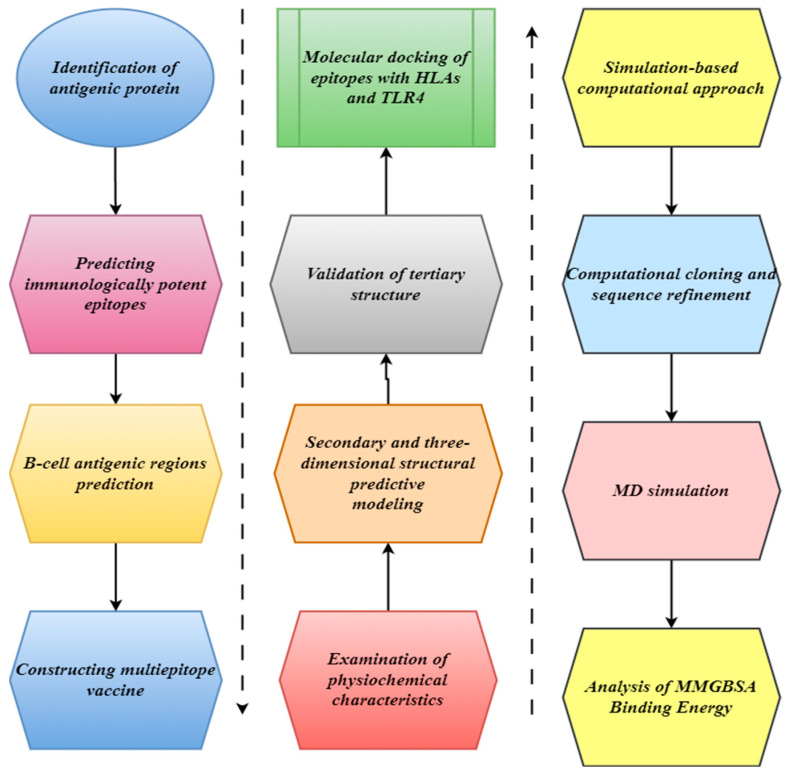
Flow chart of the current study depicting the protocol for predicting the multi-epitope vaccine construct.

The study’s flow chart is separated into many stages. First, the pathogenic viral peptides’ epitopes were predicted; subsequently, a vaccine was developed; finally, modeling and immunologic simulations were conducted, as shown in Figure 1. 

### 2.1. Identification of Antigenic Protein

The target proteome of the Bourbon virus, which consists of proteins including the nucleoprotein (460 amino acids) and polymerase subunit (PA) (636 amino acids), was provided via the NCBI database, which can be accessed at (https://www.ncbi.nlm.nih.gov/) (5 March 2024). The data were presented in the standard FASTA format. Figure 1 outlines this method’s entire workflow.

### 2.2. Predicting Immunologically Potent Epitopes

Using the NetCTL-1.2 server, the CTL epitopes inside viral proteins were predicted [22]. Additional servers, including the Net-Chop 3.1 server [23] and CTLpred [24], were used for cross-verification to guarantee correctness. The epitope prediction threshold was set to 0.75; the TAP efficiency score was set to 0.05; and the C-terminal segment breakage value was set to 0.15 by default [25]. Predictions were determined by factors such as the propensity of peptide attachment to MHC-I, proteasomal ratings near the C-cleavage and the effectiveness of antigen-related transport. Protease C-terminal cleavage score evaluation and peptide-MHC-I interaction identification were performed using artificial neural network techniques through the NetMHCpan-4.0 server. The weighted matrices approach was used to obtain the TAP performance rating. In addition, the IEDB website [26] was utilized to predict the helper T lymphocyte epitope. The highest accuracy in blind epitope prediction was ensured by employing seven distinct HLAs as a point of reference. The anticipated epitopes were given IC50 values by the IEDB servers; a smaller value indicated an increased MHC-II affinity for binding. The highest binding rates for MHC-II were shown by IC50 levels under 50 nM, medium-level affinities by rates around 500 nM and the smallest contact affinities by rates under 5000 nM.

### 2.3. B-Cell Antigenic Region Prediction

The principal functions of B cells in immunity include humoral immune response, generating memory cells to fight off subsequent infections and producing antibodies. To induce humoral immunity, one must target linear B-cell epitopes, which are antigenic areas. These epitopes, which are crucial for the development of vaccines, were predicted using the ABCpreds service [27]. To identify 20-mer continuous B-cell epitopes, these servers employ an adaptive support vector kernel approach (SVM) technique. Identifying each protein’s significant B-cell epitope based on its greatest ranking was a crucial stage in the vaccine design process.

### 2.4. Constructing a Multi-Epitope Vaccine

Several linkers were employed to join the identified epitopes: GPGPG linkers were chosen for HTL epitopes, while AAY linkers were used for CTL epitopes [28]. Additionally, the vaccine sequence was supplemented with an adjuvant named mammalian beta-defensin via an EAAAK binder at the N-terminal to improve immunogenicity. The inclusion of these linkers in the MEVC formation process stems from their critical role in facilitating epitope presentation, which in turn triggers potent immune responses. These linkers also prevent epitope folding or aggregation by maintaining the distance between the epitopes.

### 2.5. Examination of Physiochemical Characteristics

The MEVC’s antigenic potential was confirmed [29] using the Vaxijen server, and its allergenicity was predicted [30] using the Aller-TOP v. 2.0 online tool. Furthermore, the antigenic reaction of our chosen target multi-epitope vaccine design was assessed using additional adjuvants, including the TB pathogen rplL and the toxin B component of Vibrio cholerae. The several physicochemical properties of the vaccine candidate were also examined using the ProtParam web server [31]. These comprised factors such as the pI, aliphatic index and molecular weight. Furthermore, characteristics such as the susceptibility index, amino acid composition (GRAVY) and the in vitro and in vivo half-lives were measured.

### 2.6. Secondary and Three-Dimensional Structural Predictive Modeling

In order to ensure correctness in the tertiary structure, the Robbeta server [32] was applied for this purpose. Robetta uses a method known as comparative modeling to create protein structures. It locates the template structure by searching for structures corresponding to the peptide chain provided using methods such as BLAST and 3D-Jury. In the absence of a suitable template with a significant degree of similarity, Robetta employs the positioning of the de novo Rosetta module method for structure prediction. Next, using the PSI-PREDV3.3 service [33], predictions were made on the vaccine sequence’s secondary arrangement. Additionally, the online GalaxyRefine server was employed to enhance the 3D framework using techniques from CASP10 [34]. 

### 2.7. Validation of Tertiary Structure

Evaluating the tertiary atomic structure of a protein is essential. The three distinct servers were used to achieve this. The Z-score, which represents the three-dimensional quality of the amino acids’ arrangement, was computed via the ProSA web server [35]. Additionally, it provided a 3D molecular viewer with high-quality scoring plots to identify problematic regions and aid in the identification of structural elements. The protein’s 3D structure’s non-bonded interactions were calculated with the aid of the ERRAT online service [36]. Additionally, the PROCHECK service [37] was used to analyze the Ramachandran plot, which is a crucial instrument for assessing how well the arrangements of proteins are made.

### 2.8. Molecular Docking of Epitopes with HLAs and TLR4

The generation of the three-dimensional arrangements of epitopes was made simpler and more accessible by the PEP-FOLD3 approach. Whereas HLAs without crystallographic data were modeled using the Phyre2 service [38], HLAs possessing this information were retrieved via the Protein Data Bank, while the Hawkdock server [39] was used to dock selected epitopes with HLA peptides and evaluate the binding of peptides to MHC. The MM-GBSA method combines solvent-scale approaches with molecular kinetic energy to identify the best docked sites with the best binding affinities. Computational constraints and convergence problems were taken into consideration. Furthermore, the immune receptors found in immune cells have a major impact on the immune response. By employing the HDOCK web server to molecularly connect the vaccine component with the TLR-4 receptor (pdb id: 3FXI), the ability of the immunization to trigger an immune response was evaluated [40], with an emphasis on interactions between the two chains of the TLR-4 dimer.

### 2.9. Simulation-Based Computational Approach

To ascertain the impact of alien particles of antigens on the immunological systems, the web server C-ImmSim uses simulations and agent-based modeling [41]. Using the PSSM method, one may calculate the production of immunoglobulins, cytokines and interferon following vaccination and demonstrate how the antigen triggers an immunological response. It is recommended to wait four weeks between the first and second dose of most administered vaccinations. The experiment utilized time increments ranging from all parameters that stayed in their initial states from 1–84 to 8 h intervals the whole time. The two shots were administered in this arrangement four weeks apart. The Simpson index was also computed by the web server using default values, which simplified the Th1 and Th2 response predictions.

### 2.10. Computational Cloning and Sequence Refinement

Sufficient stimulation was ensured by integrating the vaccine into the host system, which was the *E. coli* strain K12. Reverse translation and codon optimization were performed in advance of expression using the JCAT online tool [42]. Codon optimization was crucial to ensuring that the vaccine expressed the vector genome as best as possible to reconcile disparities between the expression patterns of the vector genome and the genome of the Bourbon virus. When synthesizing JCat, the codon adaptation index (CAI) and GC content were considered to ensure high levels of protein production. The vaccine construct sequence was inserted into *E. coli* pET-28a(β) plasmid, with selection of restriction sites, i.e., XhoI and NdeI at the N- and C-termini, respectively, to check the expression level of our inserted sequence. Furthermore, the C-IMMSIM web server was utilized to evaluate the multi-epitope vaccination construct’s immunogenic profile. The main and secondary immune responses both have a significant impact on the immune response. The initial reaction was characterized by a high concentration of IgG + IgG and IgM. Afterward, there was an antigen decrease and the secondary and primary phases of IgM, IgG1 + IgG2 and IgG1. 

### 2.11. MD Simulation

Models were used to investigate the molecular dynamics of docked complexes. This was achieved by utilizing several components of the AMBER16 program [43]. Topological files and starting coordinates were initially created using the TLeap module with a force field ff14SB [44]; the system in the TIP3P water box with dimensions of 8.0 was solvated with proper addition of Na^+^ and Cl^−^ ions [45]. The complex’s energy was reduced by using the steepest descent to recover from unfavorable conflicts and the conjugate gradient for 1000 steps. Following ten pulses of heating, the device was monitored for temperature stability using the Langevin dynamics method. The pressure was adjusted in accordance with the procedure. In the end, the complex was subjected to an efficient simulation lasting 100 ns. By employing the standard ensembles of the simulation box, regular boundary circumstances were inferred. Berendsen coupling integration [46] was the algorithm utilized to maintain temperature stability. The TRAJectory (PTRAJ) [47] module was utilized to examine the outcomes. Xmgrace (Version 5.1.19) [48] was used to compute and show two attributes. Grace is accessible online. The gyration’s radius (RoG) and the root mean square deviation (RMSD) were utilized. It is generally accepted that an amino acid location in three dimensions is represented by alpha carbon (C) coordinates. By figuring out the average distance between them, the RMSD method examines the relative locations of protein carbon atoms over time.

### 2.12. Analysis of MMGBSA Binding Energy

The dominating simulated complexes’ binding free energies were computed using MMGBSA. The generalized Born model, molecular mechanics and solvent accessibility (MMGBSA) technique circumvents the computational difficulty of free energy simulations by extracting free energies from structural information [49]. Ante-MMPBSA.py examined and generated the first prompt files for MEBV, TLR4, MHCI, MHCII and the complexes. The variance in the receptor, complex and vaccination energies was used to derive free energy:

ΔGbind = (ΔGcomplex) − (ΔGreceptor + ΔGvaccine)(1)

The energy effects of gas-phase salvation free energy modules are shared by polar and non-polar salvation free energy modules throughout this process [49]. The following formula shows how MMGBSA computes Gibb’s free energy, a word used to express the quantity of energy, denoted by G, for each terminus:

ΔG = Egas + ΔGsolv − TS(2)

where S represents entropy, which is computed via normal mode analysis, and T stands for temperature. The force field’s MM energy is commonly used as “Egas” when it reaches the gas stage. Internal and electrostatic energy, as well as van der Waals cooperation, fall within this group.

## 3. Results

### 3.1. Protein Retrieval 

The nucleoprotein (ACCESSION ID: WLD03287) and polymerase subunit PA (ACCESSION ID: WLD03285) amino acid sequences of the Bourbon virus were obtained in FASTA format from the NCBI database. Then, using these sequences as a basis, predictions were produced regarding B- and T-cell epitopes to create a vaccination consisting of several subunits of the Bourbon virus. Before developing a vaccine, both proteins underwent screening for allergenicity and antigenicity. The findings of the analysis showed that the nucleoprotein demonstrated antigenic behavior; the Vaxijen server assigned it a score of 0.5585. Additionally, evaluations by the AllerTOP server showed that the nucleoprotein did not produce an allergic reaction. On the other hand, polymerase subunit PA likewise showed antigenicity and showed no signs of allergenic behavior, scoring significantly higher at 0.4248. All things considered, the Bourbon virus nucleoprotein and polymerase subunit PA are considered antigenic, which means that there are no allergic concerns, and they could be a good option for a multi-component subunit vaccine. 

### 3.2. Immunogenic Epitope Profiling

The NetCTL1.2 server identified 25 distinct CTL epitopes for each of the Bourbon virus nucleoprotein and polymerase subunit PA prediction analyses. These findings reflected those obtained from other epitope prediction servers, including NetChop 3.1 and CTLPred. The IEDB MHC-2 was used to identify potential (HTL) targets that were validated using a reference set of seven individual alleles. Strong binding affinity to the corresponding MHC receptors was the focus of the vaccine development process; these epitopes were identified by higher cumulative values for CTL targets alongside low percentile rankings for HTL targets. The AllerTOP and IFNepitope servers were utilized to evaluate the propensity to induce interferon (IFN) and non-overlapping sequences as selection criteria for HTL epitopes. Additionally, non-allergenicity (score below −0.4) was required. To aid in the creation of a multi-epitope vaccine, the ABCpreds server was utilized to predict LBC epitopes for the identified amino acid of the Bourbon virus. To address a variety of immune responses against the Bourbon virus, the vaccine formulation ultimately contained (6) CTL, (6) HTL and (3) B-cell epitopes per protein (Table 1). 

### 3.3. Development of a MEVC Construct 

Finally, the vaccination sequence that was created by joining distinct linkers—AAY, GPGPG and KK, respectively—included selected epitopes for B cells, the HTL and CTL. The multi-epitope vaccination was combined with an enhancer that strengthens immunity. The adjuvant, harmless human beta-defensin-2 (hBD-2), was linked with an EAAAK linker to the N-terminus of the vaccination sequence. The purpose of this modification was to improve epitope presentation on the receptors and inhibit the production of junctional epitopes. Furthermore, the antigenicity of several adjuvants was studied. With a score of 0.6810, the analysis showed that beta-defensin had a greater allergic response. This stimulates innate immunity by aiding in the activation of immune cells, such as T and dendritic cells. Its immunomodulatory properties can improve the effectiveness of the vaccination by boosting antigen presentation and inducing a potent adaptive immune response. Mammalian beta-defensin’s superb safety profile and wide range of antigen compatibility make it a fantastic choice for boosting immunization effectiveness.

**Figure 2:**
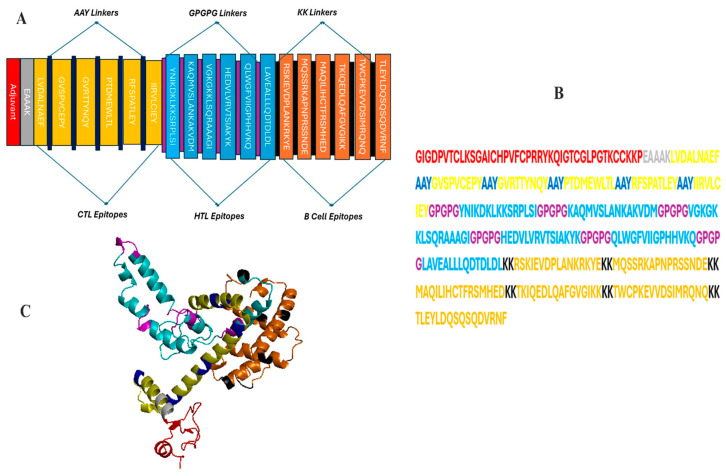
There are two distinct ways to view the final MEVC. (**A**,**B**) illustrate the arrangement of epitopes throughout the vaccine construct; (**C**) depicts the 3D model, with each component clearly colored, highlighting the vaccine folding and loop regions at N- and C-terminals.

On the other hand, the MEVC including the toxin B component of *Vibrio cholerae* and *M. tuberculosis* rpl (ribosomal protein L) demonstrated non-antigenic behavior, scoring 0.4789 and 0.4632, respectively, both below the 0.5 criterion. As shown in Figure 2**,** the final vaccination sequence included 343 amino acids, which included the epitopes. Please refer to Table 1 for additional information regarding the selected epitopes.

### 3.4. Physicochemical Properties

Analysis on the AllerTOP server, which checks allergenicity, verified the vaccine’s non-allergenic nature. Additionally, the multi-epitope vaccine (MEV) received a score of 0.7753 from the Vaxijen website, which assessed the vaccine’s antigenic potential using a virus model organism at a threshold of 0.4. The ProtParam server was utilized to calculate the vaccine’s physicochemical properties. The calculated pI was 9.55, while the molecular weight was found to be 37,504.56 Da. *E. coli* was shown to have a robust stability, with an in vivo half-life greater than 10 h. In addition, a result of the instability index—which gauges the stability of vaccines—of 43.68 was achieved (numbers below 40 are considered stable). A 75.39 aliphatic index was another indication of thermostability for the MEVC. Based on these low GRAVY values, the vaccination’s hydropathy rating was −0.469 on an overall average for hydrophilicity, and a tendency for advantageous interactions with neighboring water molecules was noted.

### 3.5. Secondary and 3D Structural Modeling

**Figure 3:**
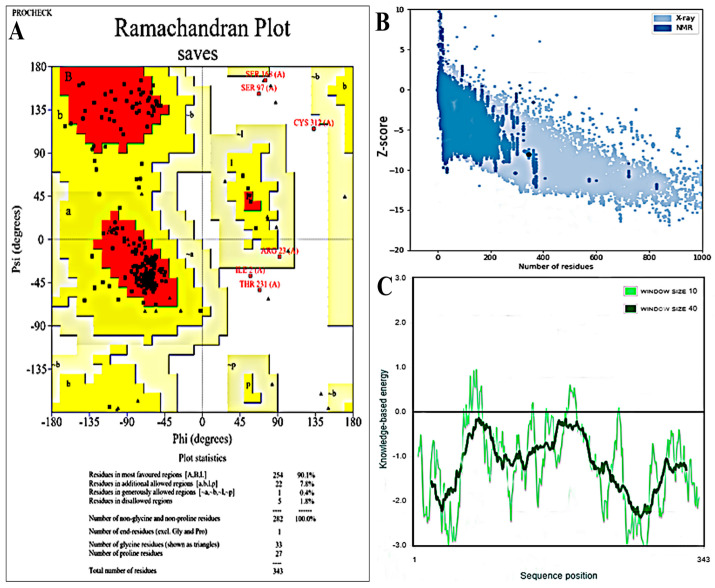
(**A**) depicts the Ramachandran plot with a confidence score; (**B**) shows the X-ray- and NMR-based prediction of the modeled vaccine construct; and (**C**) presents the sequence positioning in a 3D vaccine construct.

**Figure 4:**
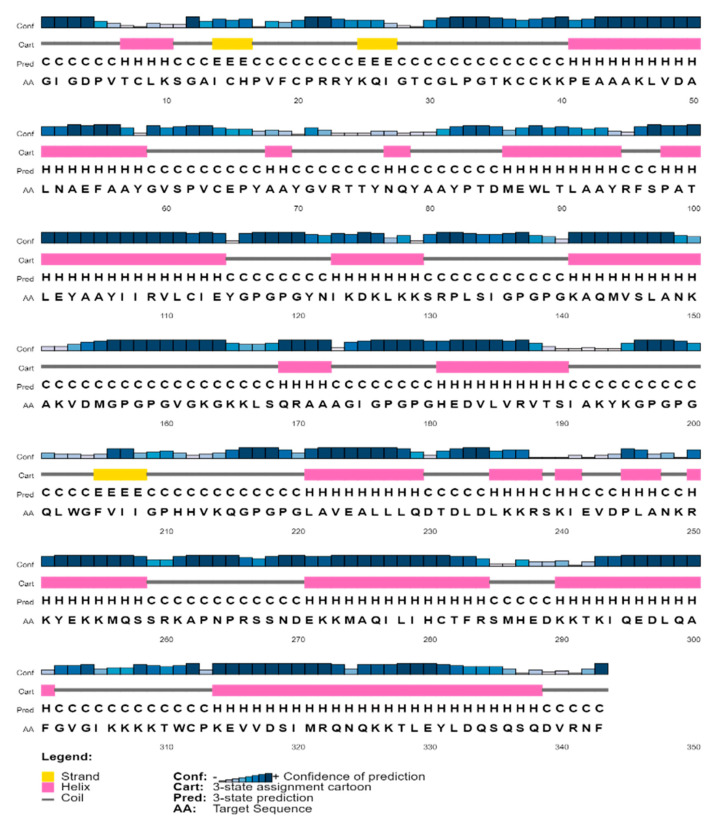
A secondary structure diagram illustrating the presence of alpha helices (46.93%), beta strands (2.9%) and coils (51%) in the vaccine design with numerous epitopes.

The PSIPRED service was used to predict the final vaccine construct’s secondary structure. Next, via a comparative modeling technique, the Robetta platform produced a 3D model for the MEVC contender. Analyses using ProSA-web, ERRAT and the Ramachandran plots were conducted to evaluate the quality of the model (Figure 3). With a Z-score of −8.03 regarding the first input model derived based on ProSA-web examination, the model is credible, since it falls within a range that is frequently observed in natural proteins with comparable sizes. A structural overall quality factor of 97.58 was also obtained through ERRAT validation. Using the Procheck server, an examination determined that 90.1% of the residues in the main model were located in the favored regions, 7.8% in the allowed regions and 1.8% in the outlier regions, according to the Ramachandran image (Figure 3A). Following a comprehensive analysis, model 1 was shown to be the most effective model, with an assurance rating of 0.46, as Robetta had predicted. The Galaxyrefine server was then utilized to improve this model structure. The server provided five models; of these, model 5 was chosen based on several criteria, including a favorable Ramachandran plot (96.2), GDT-HA (0.9942), RMSD (0.267), MolProbity (1.906), clash score (13.0) and poor rotamers (1.1). This model’s 3D representation of this choice is shown in Figure 2B. The vaccine construct structure, as shown in Figure 4, is estimated by the server to consist of 46.93% alpha-helix, 51% coil forms and 2.9% beta strands. With X-ray and NMR prediction, it was inferred that the model structure shares the best quality with a lower score at a standard level of between 5 and −10, followed by a sequence position with the knowledge-based energy level as shown in Figure 3B,C. 

### 3.6. Docking Simulations Among Peptides and MEVC

For a vaccine to be successful, it must have potent immune effects and be extremely compatible via immune-mediated receptors in the host. The three-dimensional structures of the epitopes were used with PEP-FOLD3. Most of the HLA 3D models were sourced from the Protein Data Bank (PDB), and if crystallographic data were not available, Phyre2 was employed to generate the models for individual HLAs. The Hawkdock server was used to perform HLA-peptide docking to evaluate peptide–MHC interactions. The MM-GBSA was used to calculate the bonding kinetic energy methodology, which combines molecular mechanics energies with solvent accessibility and generalized Born methods. The entropy component was excluded in certain circumstances due to difficulties with convergence, and its computation proved impracticable in other cases.

**Figure 5:**
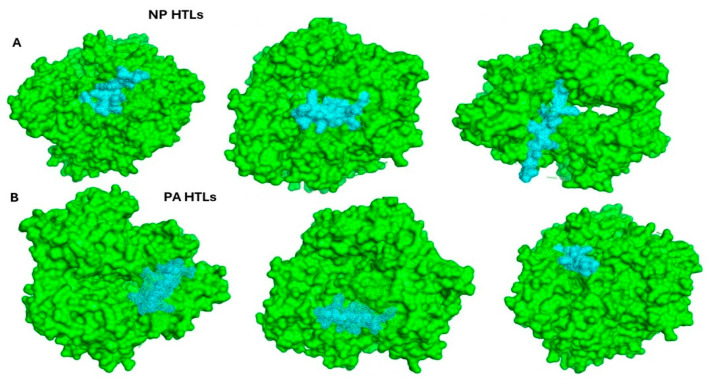
(**A**) depicts the binding pose of epitopes against nucleoprotein HTLs; (**B**) presents the binding pose of polymerase subunit PA HTLs in complex with epitopes.

**Figure 6:**
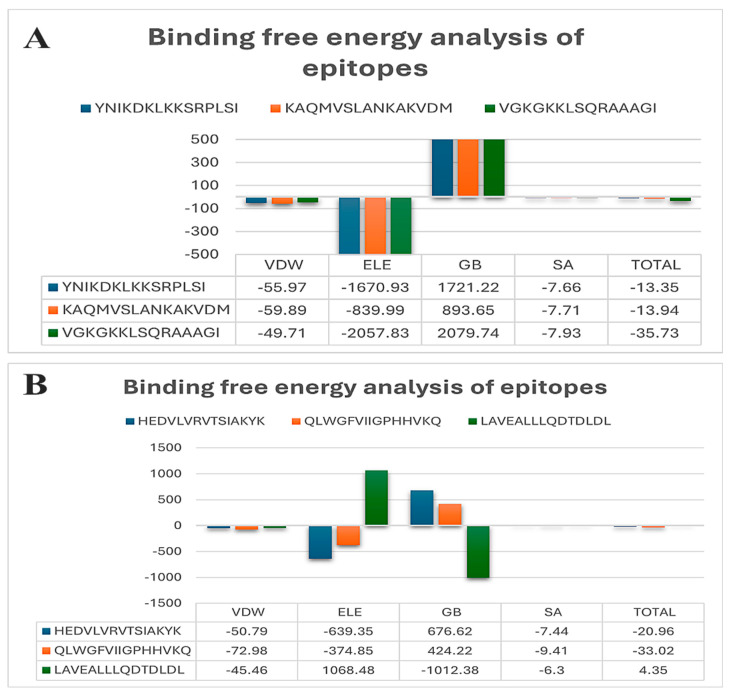
(**A**) shows the graphical insights of binding free energies for epitopes against nucleoprotein HTLs; (**B**) illustrates the binding free energies’ calculation of polymerase subunit PA HTLs in complex with the predicted epitopes.

The overall binding free energy for the nucleoprotein (NP) HTL in complex with epitopes obtained during docking showed promising outcomes. Herein, we found an epitope, i.e., YNIKDKLKKSRPLSI, in complex with hLA-DRB1*15:01, with PDB id: 8TBP, showing a binding affinity of −13.35 kcal/mol, whereas the epitope, i.e., KAQMVSLANKAKVDM, in complex with hLA-DRB1*15:01 with PDB id: 8TBP resulted in −13.94 kcal/mol, and the third epitope, i.e., VGKGKKLSQRAAAGI, in complex with hLA-DRB1*15:01 with PDB id: 8TBP showed a low and stable binding affinity of −35.73 kcal/mol (Figure 5A). On the other hand, the energies for the HTL epitopes of the polymerase subunit PA also resulted in stable and low binding energies. Herein, we calculated −20.96 kcal/mol for HEDVLVRVTSIAKYK-hLA-DRB1*15:01 with PDB id: 8TBP, −33.02 kcal/mol for the QLWGFVIIGPHHVKQ-hLA-DRB1*15:01 complex with PDB id: 8TBP, and finally, 4.35 kcal/mol for the LAVEALLLQDTDLDL-hLA-DRB1*15:01 complex with PDB id: 8TBP (Figure 5B). Additionally, the estimated bonding kinetic energy for the docked peptide-HLA clusters and the complexes themselves are shown in Figure 6, respectively.

### 3.7. Codon Optimization of the Comple Vaccine Construct 

**Figure 7:**
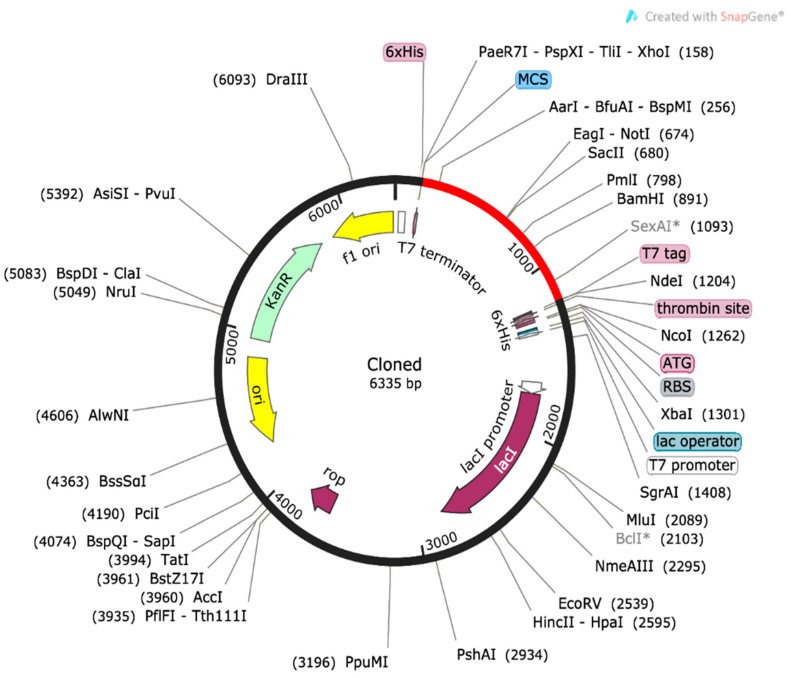
The red line, surrounded by a black circle, symbolizes the MEV’s restriction cloned into the pET28a(+) transcription vector in silico.

The synthesis of the vaccine protein was optimized using the Java Codon Adaptation Tool to improve codon use compatibility with *Escherichia coli* (strain K12). After optimization, the sequence with 1035 nucleotides had a GC content of 55.85% and a CAI of 0.94. As the GC percentage fell within the recommended range of 30–70%, the circumstances were suitable for producing the vaccine protein in *E. coli*, according to the data. The restriction clone was then created by adding the modified codon sequences to the pET28a (+) vector, as seen in Figure 7.

### 3.8. Immune Simulation

**Figure 8:**
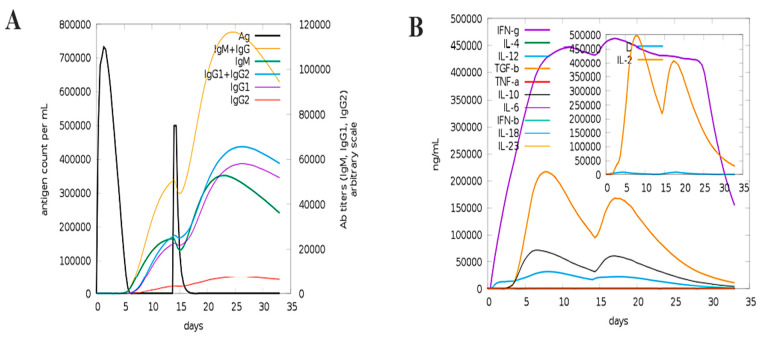
MEBV in silico cloning and immune simulation investigations. (**A**) The amount of interleukin and interferon produced in milliliters per nanogram in response to MEBV. (**B**) The immunoglobulin response measured in microliters in response to the MEBV antigen.

To evaluate the effect of immunization, a host immune system simulator reaction was conducted four weeks apart after the administration of first and second doses. As shown in Figure 8A**,** the simulated immune response closely resembled observations of usual immunological reactions in comparable settings. Notably, compared to the main response, the secondary response (occurring after the second dose) showed a noticeably stronger reaction. 

The first phase of the immunological response was characterized by elevated IgM antibody levels. Following this, there was a clear decrease in antigen concentrations and a rise in antibody levels (IgM, IgG + IgM and a mix of IgG1/IgG2 autoantibodies) in both the primary and secondary responses. The reaction of cytokines and interleukin was strong, as indicated by increased IFN-γ and IL-2 levels. Moreover, as shown in Figure 8B, there was a noteworthy decline in the Simpson index (D), suggesting increased diversity within the immune system. This profile of immunological simulation points to the development of immune memory and possible protection against the Bourbon virus.

### 3.9. Molecular Docking

**Figure 9:**
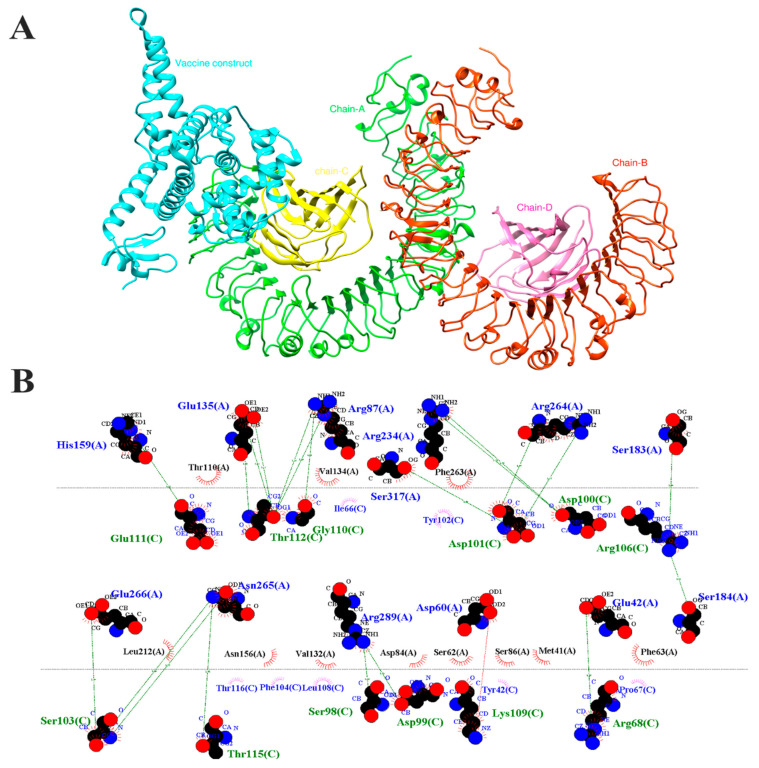
(**A**) TLR-4 receptor and vaccine with docked pose. The vaccine construct is shown in cyan and is also encoded by (C)**,** whereas the TLR-4 receptor networks A, B, C and D are colored green, red, yellow and pink, respectively. (**B**) shows the receptor’s interaction residues with vaccine construct encoded by (C).

The vaccination construct and the TLR4 human receptor were molecularly docked using the HDOCK service. The server generated ten clusters and found the membership count of each cluster with the lowest energy value. After comparing all clusters, it was found that cluster 1 had the largest negative interaction energy and was the one with the smallest number of −678.8 kcal/mol (Figure 9A). The LigPlot tool demonstrated the connections that were made between TLR4 and the amino acids in the vaccine construct (Figure 9B). The vaccine and the receptor with the highest binding affinities are indicated by the lowest global energy. The residues of the vaccine construct included His159A-Glu111C, Glu135A-Thr112C, Glu135A-Gly110c, Arg87A-Thr112C, Arg87A-Gly110C, Ser317A-Asp101C, Arg234A-Asp110C, Arg264A-Asp101C, Ser183A-Arg106C, Glu266A-Ser103C, Asn265A-Thr115C, Arg289A-Ser98C, Arg289A-Asp99, Asp60A-Lys109C and Glu42A-Arg68C, and residues from the TLR4 chain A were involved in both hydrophilic and hydrophobic interactions within a 3Å distance, as shown in Figure 9B. For additional stability analysis, the molecular dynamics simulation was applied to the chosen docked complex.

### 3.10. MD Simulation 

**Figure 10:**
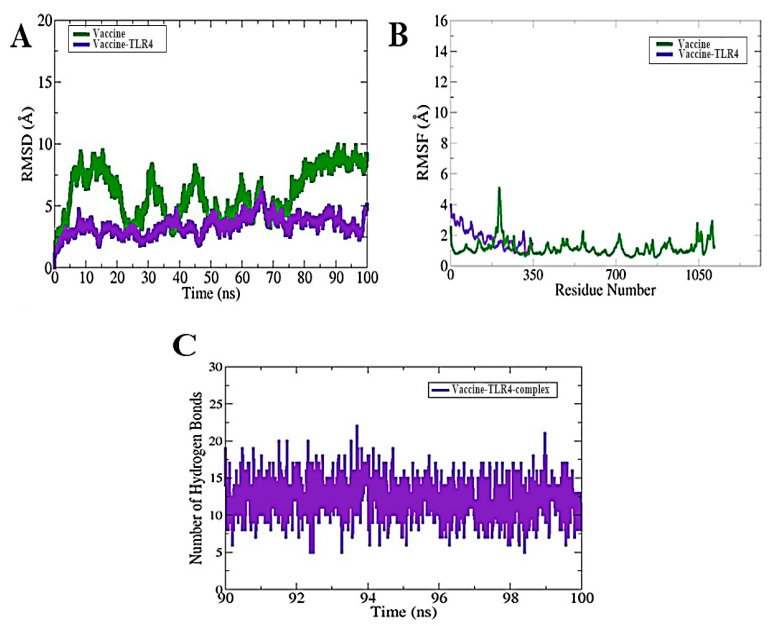
(**A**) An examination of the vaccine–TLR4 complexes and vaccine RMSD graphs at 100 ns time intervals. (**B**) The vaccine–TLR complexes’ RMSF plot. (**C**) The vaccine–TLR complexes’ H-bonds plot.

To investigate the atomic-level physical motions and verify the stability of the docked complex, a molecular dynamics simulation was run. To measure the complex’s dynamic behavior and stability throughout a 100 ns run, the root mean square deviation and root mean square fluctuation were calculated along with hydrogen bond analysis. The results show that the graph first fluctuated at 3 Å at 2 ns and increased to 4.9 Å (Figure 10A). The root mean square deviation (RMSD) shows a distance to the backbone carbon alpha of stacked proteins. The system stabilized around 10–68 ns but had some significant oscillations between 65 and 70 ns (Figure 10A). It then re-equilibrated and became stable until the end of the simulation time intervals, with an average root mean square deviation of 3.6 Å. An RMSD for the vaccine construct was also calculated, which showed fluctuation at certain time intervals. The to-and-fro motion was reduced within 10–65 ns but remained stable until the end. The vaccine remained intact, with the chain C and chain A of the TLR4 receptor with an average RMSD of 5.8 Å (Figure 10A). 

To look at the shift in the vaccine–TLR complexes’ structural flexibility at the residue level, root mean square fluctuation (RMSF) was computed. The RMSF values of the vaccine–TLR4 complex ranged from 1.35 to 2.62 Å, but the vaccine–TLR4 complex’s RMSF plot showed notable variations, with the lowest and highest values being 1.25 and 5.25 Å, respectively (Figure 10B). A key factor in the stability of proteins is the presence of intermolecular hydrogen bonds, or H-bonds. A maximum of 18 H-bonds were created by the vaccine–TLR4 complex and a maximum of 22 H-bonds by the vaccine–TLR4 complex with a time interval of 100 ns (Figure 10C). 

### 3.11. Binding Free Energy Calculations

In biomolecular research like protein–protein interactions, the most often used and favored methods for determining the binding free energy are the MMPB/GB-SA approaches. The free energy perturbation technique is significantly more cost-effective than these approaches, which are also less precise than docking/scoring methods. Table 2 lists the binding energies of the system under study using both MMPBSA approaches. A net binding energy of −134.83 kcal/mol in MMGBSA demonstrated the system’s strong and stable binding. A gas-phase energy of −166.25 kcal/mol dominated this energy in complex systems. Van der Waals energy (−120.56 kcal/mol) contributed less to the gas-phase energy. The total solvation energy recorded was 31.42 kcal/mol, along with Columbic energies of −45.69 kcal/mol. Thus, these binding energies contributed to a highly stable environment and making the system stable throughout the time intervals.

## 4. Discussion

Immunoinformatic techniques have shown to be quite successful when it comes to creating cutting-edge multi-epitope vaccine candidates to fight a variety of dangerous diseases, such as infections brought on by the Bourbon, Colorado tick fever, dengue and other viruses [26]. Thanks to epitopes, these strategies have become increasingly popular in the field of vaccine development. The high death rate linked to viruses like the Bourbon virus and the lack of available treatments have hindered efforts worldwide to produce vaccines utilizing full or partial parts of viral proteins [50].

Additionally, the critical functions that the nucleoprotein and polymerase subunit PA play in virus entry and packaging into host cells led to their selection. It has been determined that structural proteins are important initiators of the host immune response [51]. One of the most useful, successful and economical strategies for enhancing public health and battling infectious diseases is vaccination. In today’s world, it is considered one of the safest and most successful treatments. Because of their distinct immunogenic components, subunit vaccinations are becoming more and more popular compared to whole-pathogen vaccines [52]. Extensive proteome and genomic information on a range of dangerous microbes, such as the Bourbon virus, has become easily accessible on the internet in recent years. These data sources have made a substantial contribution to the advancement of vaccine development research [26,53]. 

The discovery of novel antigenic targets is facilitated by the compilation of data using computer technologies. Understanding the interactions between cells, host MHC alleles and Bourbon virus protein epitopes holds enormous immunological promise. Examining the immune system reaction that these interactions produce will make developing a vaccine candidate to combat the Bourbon virus easier. This investigation involved a thorough analysis of two Bourbon virus proteins. To ensure that epitope-driven vaccines would not cause autoimmune reactions, the BLASTp analysis was used to compare the human proteomes with these target viral proteins. It was anticipated that T and B cells would be able to identify epitopes on the viral proteins. T lymphocytes can selectively identify MHC molecules through endocytic processes, and on the outer peptide of T-lymphocyte receptors, these molecules often assist CD4+ T cells. Immunoinformatic analysis was used to identify several MHC class I and II and B-cell epitopes based on unique structural and physiochemical characteristics in our final vaccine design. Because it lacked allergies and exhibited strong antigenicity, the resulting multi-epitope subunit vaccine protein had potential as a vaccine candidate. At 37,504.56 kDa, the vaccine protein satisfies the criteria outlined in the standard processes for producing multi-epitope vaccination candidates (MEVCs). The protein’s theoretical pI of 9.55 demonstrates its fundamental characteristics, and the aliphatic index verifies that aliphatic side chains are present within the protein structure. Additionally, the vaccine protein’s strong stability, as shown by the instability score, suggests that it is resistant to losing its structural integrity when heated. This study examined the vaccine’s secondary structure using PSIPRED V3.3, providing valuable insights on its structural features. The three-dimensional structure was also generated using homology modeling, which facilitated the investigation of protein behavior, activity and interactions with ligands and other proteins, as well as the clarification of the spatial arrangement of essential protein residues. The completed vaccination model underwent structural validation procedures to guarantee its dependability. The main Ramachandran plot showed that most residues were located in the favored region, indicating a satisfactory level of overall reliability. The immune system’s response was evaluated by molecular docking between the vaccination and TLR-4; immunostimulant studies predicted a response in line with typical immunological patterns. Notably, following two doses separated by four weeks, there was a notable improvement in immunological responses, as indicated by the release of IgM and IgG, which was followed by sustained levels of IgM, IgG1 and IgG2, as well as a reduction in antigen levels. Increased IFN-γ and IL-2 concentrations bolstered successful immune responses even more. *E. coli* (strain K12) produced more of the recombinant vaccine protein because of codon optimization, which improved transcription and translation efficiency. When overexpressed, the vaccine showed good solubility, which correlated with greater protein robustness. High binding affinities were found using molecular docking techniques, and these findings were supported by molecular dynamics simulations, which showed stable interactions with hydrogen bonds and low-level energy states. As a result of these interactions, the provided epitopic antigens trigger both an adaptive and a naïve immune response. Strong connections between MEBV and TLR4, MHC I, and MHC II were validated via molecular docking and the MD modeling process, and the MMGBSA experiments showed that this stable bonding required very little energy. During MD simulations, there were slight variations and a considerable amount of H-bonds during docking. These results demonstrate the effectiveness of the MEBV’s immune receptor binding. Although strong immune responses were noted in the experimental animal models, the complexity of immunopathology makes it difficult to apply these findings to human testing. Confirming the vaccine’s safety and efficacy in preventing Bourbon virus infection will require additional testing, such as larger animal models or human trials. Further research is necessary to determine whether the vaccine is suitable for broad use, including a review of any possible side effects and the long-term stability of immune responses. This work attempts to produce a potentially safe and effective vaccination against Bourbon virus infection by applying modern immunoinformatic techniques. 

Although the study’s findings are promising, certain limitations of the work must nevertheless be addressed in the future. Experimental validation of the immune protection efficacy of the designed vaccine construct must be performed in in vitro and in vivo assays. Further, the predicted antigenic epitopes’ ordering in the designed vaccine construct must be examined to achieve the best ordering for maximum protective immunity. Additionally, predictions regarding the selected epitopes’ interactions with the MHC molecules must be checked. Thus, to improve the development of the proposed vaccine, proper experiments must be pipelined. Plaque reduction neutralization tests (PRNTs) should be used during in vitro experiments to evaluate the ability of the vaccine construct to elicit neutralizing antibodies [54]. Antigen presentation studies using dendritic cells should also be used to evaluate the degree to which the predicted antigenic epitopes activate T cells [55]. Furthermore, the immune response may be understood by assessing cytotoxic T-cell activity in co-culture systems [56]. To test the effectiveness of vaccines against the Bourbon virus, pathogen challenge investigations and assessments of humoral and cellular immunity should be carried out in mice or non-human primates, which are suitable animal models for in vivo assays [57]. The use of methods such as ELISPOT and flow cytometry in longitudinal immune profiling will enable researchers to track alterations in immune responses over time [58,59]. By highlighting the multi-disciplinary character of vaccine research, this extensive framework for further experimental work will greatly involve the bioengineering researchers. Secondly, it is important to note that there is a lack of clarity regarding the transfer of vaccine efficacy from mouse research to human use. To determine the best immunization schedule for humans in terms of safety and protective effectiveness, further research on vaccine efficacy in more clinically relevant animal models, such as non-human primates, is required.

## 5. Conclusions

The Bourbon virus was initially discovered in a patient from Kansas’s Bourbon County in 2014. The patient’s severe illness, which led to death, was connected with problems resulting from the high rate of infection. Tick bites are the main mechanism through which the Bourbon virus spreads, with other routes of transmission also possible. Fever, exhaustion, rash and other flu-like symptoms are common signs of infection; in severe cases, multi-organ failure and death may result. There are currently no antiviral medications or vaccines specifically designed to treat the Bourbon virus, which emphasizes the need for continued research to combat this newly discovered infectious disease. Thus, we investigated a highly antigenic protein target utilizing a variety of computing and reverse vaccinology methods. The research team predicted T- and B-cell epitopes within two essential Bourbon virus proteins—the nucleoprotein and the polymerase subunit PA—with a non-allergenic, highly immunogenic, non-toxic, multi-epitope vaccine construct. Furthermore, a molecular dynamic simulation study validated the vaccine construct against a target TLR-4 human receptor in a real-time environment. Thus, such an integrated vaccinomics strategy opens the gateway for future researchers to explore it during pharmacological trials to further strengthen its efficacy. 

## Figures and Tables

**Table 1:** (A) depicts the cytotoxic T-cell epitope selection; (B) shows the helper T-cell epitope selection; meanwhile, (C) shows the B-cell epitopes selected.

(A)						
Protein	Peptide	Affinity	Cleavage	Tap Score		Combined Score
Nucleoprotein	LVDALNAEF	0.2994	0.9286	2.5550		1.5382
	GVSPVCEPY	0.1263	0.9660	2.9280		0.8276
	GVRTTYNQY	0.1057	0.9748	3.0650		0.7483
Polymerase subunit PA	PTDMEWLTL	0.2933	0.8914	0.4090		1.3995
	RFSPATLEY	0.1405	0.9767	3.3020		0.9083
	IIRVLCIEY	0.0817	0.7837	3.0690		0.6178
**(B)**						
**Protein**	**Allele**	**Peptide**		**Score**	**Allergenicity**	**IFN**
Nucleoprotein	HLA-DRB1*15:01	YNIKDKLKKSRPLSI		1.22	Nill	**Yes**
	HLA-DRB1*15:01	KAQMVSLANKAKVDM		0.34	Nill	Yes
	HLA-DRB1*15:01	VGKGKKLSQRAAAGI		0.25	Nill	Yes
Polymerase subunit PA	HLA-DRB1*15:01	HEDVLVRVTSIAKYK		0.53	Nill	Yes
	HLA-DRB1*15:01	QLWGFVIIGPHHVKQ		0.67	Nill	Yes
	HLA-DRB1*15:01	LAVEALLLQDTDLDL		1.78	Nill	Yes
**(C)**						
**Protein**	**Position**	**Peptide**		**Score**		
Nucleoprotein	24	RSKIEVDPLANKRKYE		0.91		
	1	MQSSRKAPNPRSSNDE		0.91		
	307	MAQILIHCTFRSMHED		0.85		
Polymerase subunit PA	295	TKIQEDLQAFGVGIKK		0.87		
	62	TWCPKEVVDSIMRQNQ		0.85		
	132	TLEYLDQSQSQDVRNF		0.83		

**Table 2:** Binding free energies’ calculation of the MEVC with the TLR-4 receptor after simulation time intervals of 100 ns.

Energy Parameter	Vaccine–TLR4 Complex
Van der Waals Energy (kcal/mol)	−120.56
Columbic Energy (kcal/mol)	−45.69
Total Gas-Phase Energy (kcal/mol)	−166.25
Total Solvation Energy (kcal/mol)	31.42
Net Energy (kcal/mol)	−134.83

## Data Availability

The data generated in this study is presented in this paper.
